# Design, synthesis and therapeutic potential of 3-(2-(1*H*-benzo[*d*]imidazol-2-ylthio)acetamido)-*N*-(substituted phenyl)benzamide analogues

**DOI:** 10.1186/s13065-018-0513-3

**Published:** 2018-12-19

**Authors:** Sumit Tahlan, Kalavathy Ramasamy, Siong Meng Lim, Syed Adnan Ali Shah, Vasudevan Mani, Balasubramanian Narasimhan

**Affiliations:** 1 Faculty of Pharmaceutical Sciences, Maharshi Dayanand University, Rohtak, 124001 India; 2 Faculty of Pharmacy, Universiti Teknologi MARA (UiTM), 42300 Bandar Puncak Alam, Selangor Darul Ehsan Malaysia; 3 Collaborative Drug Discovery Research (CDDR) Group, Pharmaceutical Life Sciences Community of Research, Universiti Teknologi MARA (UiTM), 40450 Shah Alam, Selangor Darul Ehsan Malaysia; 4 Atta-ur-Rahman Institute for Natural Products Discovery (AuRIns), Universiti Teknologi MARA (UiTM), Puncak Alam Campus, 42300 Bandar Puncak Alam, Selangor Darul Ehsan Malaysia; 5 Department of Pharmacology and Toxicology, College of Pharmacy, Qassim University, Buraidah, 51452 Kingdom of Saudi Arabia

**Keywords:** *m*-Amino benzoic acid, 2-Mercaptobenzimidazole, Benzamide, Antibacterial, Antifungal, Anticancer, SAR

## Abstract

**Background:**

The emergence of bacterial resistance is a major public health problem. It is essential to develop and synthesize new therapeutic agents with better activity. The mode of actions of certain newly developed antimicrobial agents, however, exhibited very limited effect in treating life threatening systemic infections. Therefore, the advancement of multi-potent and efficient antimicrobial agents is crucial to overcome the increased multi-drug resistance of bacteria and fungi. Cancer, which remains as one of the primary causes of deaths and is commonly treated by chemotherapeutic agents, is also in need of novel and efficacious agents to treat resistant cases. As such, a sequence of novel substituted benzamides was designed, synthesized and evaluated for their antimicrobial and anticancer activities.

**Methodology:**

All synthesized compounds were characterized by IR, NMR, Mass and elemental analysis followed by in vitro antimicrobial studies against Gram-positive (*Staphylococcus aureus*), Gram-negative (*Salmonella typhi* and *Klebsiella pneumoniae*) bacterial and fungal (*Candida albicans* and *Aspergillus niger*) strains by the tube dilution method. The in vitro anticancer evaluation was carried out against the human colorectal carcinoma cell line (HCT116), using the Sulforhodamine B assay.

**Results, discussion and conclusion:**

Compound **W6** (MIC_*sa, st, kp*_ = 5.19 µM) emerged as a significant antibacterial agent against all tested bacterial strains *i.e.* Gram-positive (*S. aureus*), Gram-negative (*S. typhi, K. pneumoniae*) while compound **W1** (MIC_*ca, an*_ = 5.08 µM) was most potent against fungal strains (*A. niger* and *C. albicans*) and comparable to fluconazole (MIC = 8.16 µM). The anticancer screening demonstrated that compound **W17** (IC_50_ = 4.12 µM) was most potent amongst the synthesized  compounds and also more potent than the standard drug 5-FU (IC_50_ = 7.69 µM). 
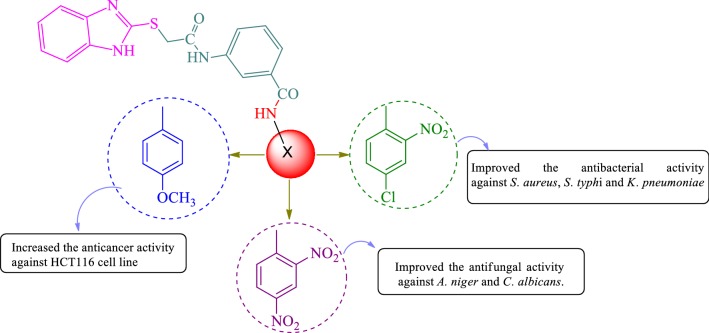

## Background

Antibiotics with a wide spectrum of activity are considered to be most potent against Gram positive or Gram-negative microbes. Nevertheless, there is now a frightening increment of resistance against commercially accessible antimicrobials which diminishes the scope of treatment against distinctive irresistible infections. Resistance against *β*-lactam, macrolides, quinolones, and vancomycin is amongst the most vital medical issues. This has called for the discovery of novel and potent antimicrobials with varying chemical characteristics [1].

Colorectal tumour (CRC) is one of the most widely recognized gastrointestinal malignancies. Changes in way of life, high-fat eating regimen, physical apathy and smoking are related to CRC pathogenesis. About 25% instances of CRC were presented with metastases at early analysis and nearly 50% of CRC patients would suffer from metastasis at some stage of life. To a large extent, the outcomes of treatment for these patients are unsatisfactory because usual regimens consider the probability of homogeneous distribution of tumor mass. It is increasingly recognized that CRC is sustained by a distinctive set of neoplastic cells named “cancer stem cells (CSCs)” which have an intrinsic potential of stemness (protection from treatment) and oncogenesis (malignant growth) [2].

Owing to their diverse potential, heterocyclic scaffolds are of major interest in the pharmaceutical industry. The heterocyclic entities which have a nitrogen group occupying a central position make them highly resemblance in structure with numerous naturally occurring molecules. In terms of improvement of biologically and therapeutically important molecules, benzimidazole nucleus has been proven as the most advantageous pharmacophore [3].

**Fig. 1:**
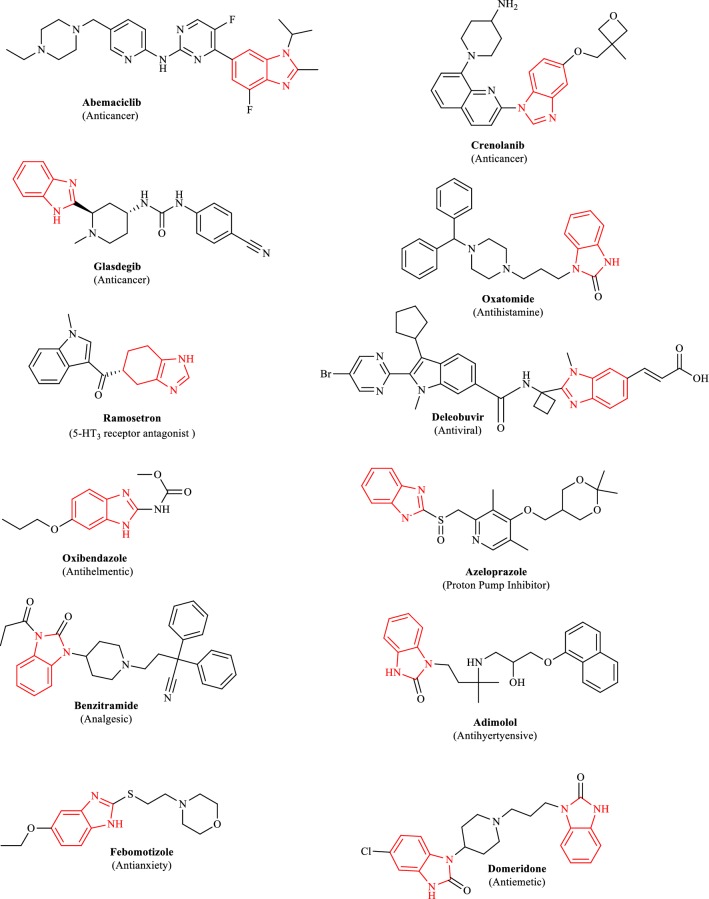
Marketed medicines containing benzimidazole as core moiety

Several benzimidazole motifs with wide spectrum of diversified biological and pharmacological potentials have already been published in literature. They were reported to exhibit essential antimicrobial and antifungal [4–7], anticancer [8, 9], antiulcer [10], antihistaminic [11], antiviral [12], antihelmentic [13], analgesic [14], antihypertensive [15] and antidepressant activities [16]. Figure 1 shows a profound number of marketed medicines containing benzimidazole as a core moiety. There is now a profoundly large number of benzimidazole at several stages of evaluation in different research associations all over the globe [17].

**Fig. 2:**
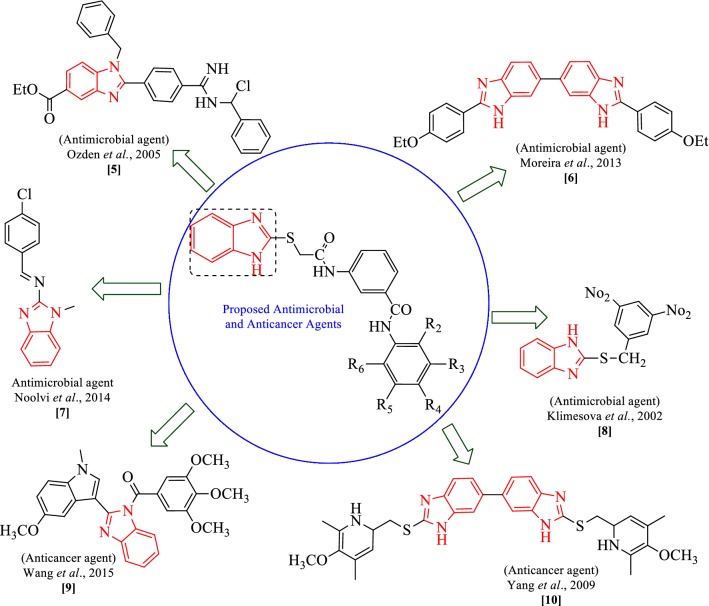
Design of benzimidazole analogues for antimicrobial and anticancer activity based on biological profile

Benzimidazole is also a vital pharmacophore, a privileged sub-structure in medicinal chemistry which contributes as a key part for different natural activities. The prominent organic applications as demonstrated by molecules related to these cores have incited wide examinations for their synthesis. Extensive biochemical and pharmacological investigations have affirmed that benzimidazole derivatives are effective against different microorganisms. Given the basic resemblance with purine, antibacterial capacity of these scaffolds showed that their opposition with purines would bring about a particular hindrance against the combination of nucleic acids and proteins inside the bacterial cell wall [18, 19]. Figure 2 reviews the biological and therapeutic profile of benzimidazoles as motivated by the structural features. As part of our continued effort in exploring new therapeutic molecules, we hereby report the synthesis, antimicrobial and anticancer evaluation as well as SAR studies of some 3-(2-(1*H*-benzo[*d*]imidazol-2-ylthio)acetamido)-*N*-(substituted phenyl) benzamide derivatives.

## Results and discussion

### Chemistry

**Scheme 1:**
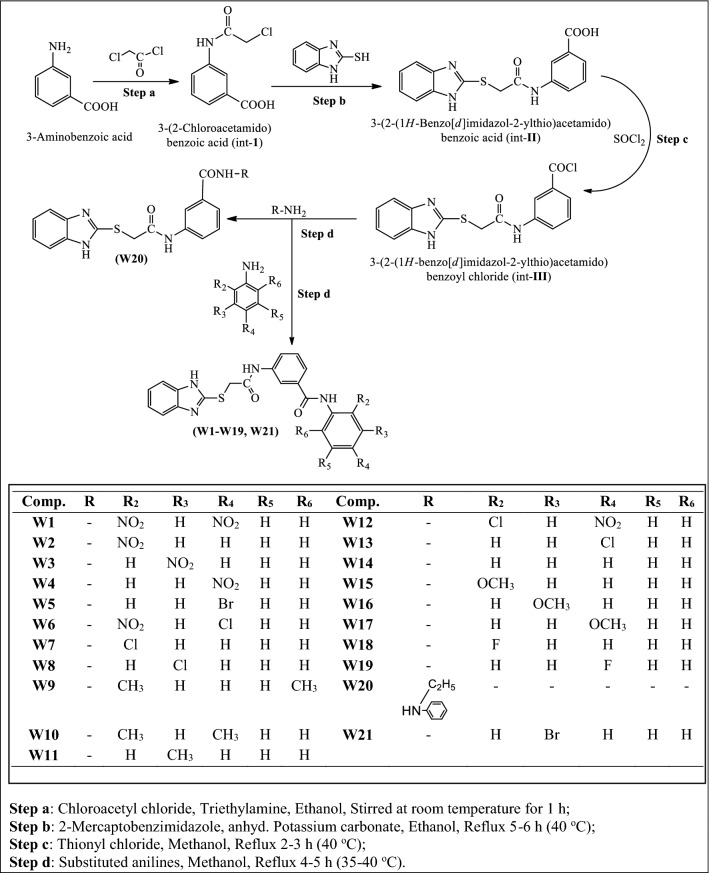
Synthesis of 3-(2-(1*H*-benzo[*d*]imidazol-2-ylthio)acetamido)-*N*-(substitutedphenyl) benzamides

The new target molecules **(W1–W21)** had been synthesized by multistep procedure as discussed in Scheme 1. Firstly, the 3-(2-chloroacetamido) benzoic acid **(**int**-I)** was synthesized by the reaction of chloroacetyl chloride with *m*-amino benzoic acid, then it was refluxed with 2-mercaptobenzimidazole which yielded 3-(2-(1*H*-benzo[*d*]imidazol-2-ylthio)acetamido)benzoic acid **(**int**-II)** which on reaction with thionyl chloride in suitable alcohol resulted in formation of 3-(2-(1*H*-benzo[*d*]imidazol-2-ylthio)acetamido)benzoyl chloride **(**int**-III)**. The reaction of above synthesized benzoyl chloride **(**int**-III)** with different substituted anilines in methanolic/ethanolic solvent yielded the title scaffolds **(W1–W21)** with appreciable yields. The physicochemical properties with Mass spectra and elemental analysis of synthesized compounds are given in experimental section. The synthesized molecules **(W1–W21)** were also illustrated by FT-IR, proton and carbon-NMR data which are in concurrence with the proposed molecular structures of synthesized compounds. The IR stretching vibration at 3107–3097 cm^−1^ and ~ 1600 cm^−1^ illustrated the occurrence of aromatic C–H and C = C groups, respectively. The IR band in the range of 653 cm^−1^ to 651 cm^−1^ corresponds to the C–Br stretching of Ar-Br compounds (**W5** and **W21)**. The presence of aromatic nitro group in compounds, **W1, W2, W3, W4, W6** and **W12** is indicated by the appearance of stretching in the range of 1554–1484 cm^−1^. IR band appearance around 2832–2820 cm^−1^ has established the existence of Ar-OCH_3_ (an arylalkyl ether) in compounds **W15**, **W16** and **W17**. Moreover, halogen group presence in compounds, **W6**, **W12** and **W13** is specified by the presence of stretching vibration at 758–742 cm^−1^ of Ar–Cl and in compounds **W18** and **W19** by presence of Ar-F stretching at 1085–1084 cm^−1^. IR stretching at 2900–2869 cm^−1^ in the spectral data of synthesized derivatives (**W9**–**W11**) depicted the presence of Ar-CH_3_. Presence of C–S group is indicated by the appearance IR stretching at 708–679 cm^−1^ in synthesized compound’s spectral data. The appearance of –CONH– group is suggested by the IR stretching at 1670–1662 cm^−1^ spectral data of synthesized compounds. The presence of –C=N– (3^o^ amine) and –C-NH– (2^o^ amine) groups is suggested by the appearance of IR stretching 1372–1321 cm^−1^ and 1338–1302 cm^−1^ of synthesized compound’s spectral data respectively. The structures of *N*-phenylbenzamide were further confirmed by the corresponding ^1^H-NMR. The multiplet signals between 7.12 and 10.75 δ ppm in ^1^H-NMR spectra indicated the aromatic proton of synthesized derivatives. The presence of –CH_2_– and –CONH– between 2-mercaptobenzimidazole and *para* amino benzoic acid in all the synthesized derivatives were indicated by appearance of singlet at 2.51–4.34 δ ppm and 7.82–8.15 δ ppm, respectively. Owing to presence of CH_3_ of Ar-CH_3_, the compounds **W9**, **W10** and **W11** reflected singlet at 2.51–2.53 δ ppm. As a result of presence of –OCH_3_ of Ar-OCH_3_, compounds **W15**, **W16** and **W17** showed singlet at range of 3.73–3.74 δ ppm. Due to presence of -NH- of benzimidazole, all synthesized compounds reflected singlet at 4.31–4.37 δ ppm. The findings of elemental analysis of synthesized 2-mercaptobenzimidazoles were recorded within theoretical results of ± 0.4%. Conclusively, the ^13^C-NMR spectra of synthesized benzamides were in DMSO-*d6* and their molecular structures were in accordance with the spectral signals. Mass spectra of the synthesized derivatives reflected the characteristic molecular ion peaks.

### In vitro antimicrobial and anticancer screening results

**Fig. 3:**
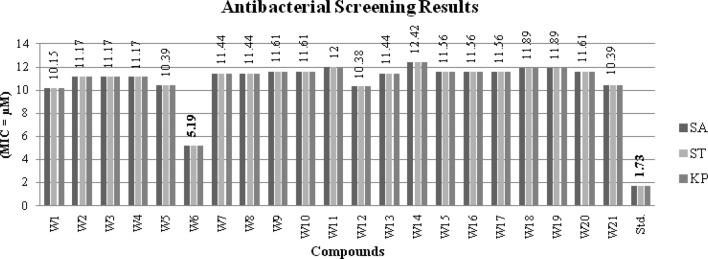
Antibacterial screening results against Gram positive and Gram negative species

**Fig. 4:**
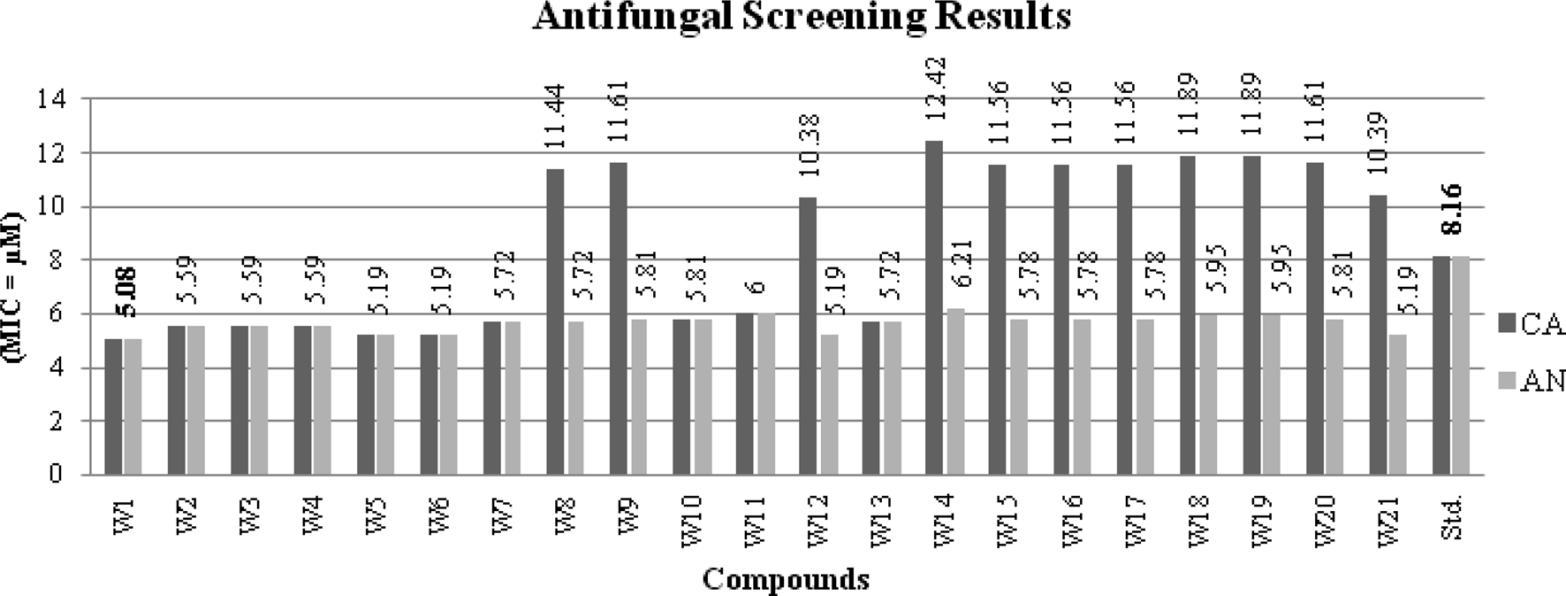
Antifungal screening results against fungal species

The antimicrobial results of synthesized of benzamides (**W1–W21**) are presented in Table 1, Figs. 3 and 4. The antimicrobial potential of synthesized compounds is comparable to the reference ofloxacin (antibacterial) and fluconazole (antifungal). Antimicrobial screening results revealed that among the synthesized compounds, 4-chloro-2-nitro substituted benzamide i.e. compound **W6** (MIC_*sa, st, kp*_ = 5.19 µM) exhibited significant antibacterial potency against all the tested bacterial strains i.e. Gram-positive (*S. aureus*), Gram-negative (*S. typhi, K. pneumoniae*) while the antifungal results indicated that the 2, 4-dinitro substituted derivative i.e. compound **W1** (MIC_*ca, an*_ = 5.08 µM) was found to be most potent against fungal strains (*A. niger* and *C. albicans*), even more potent than standard fluconazole (MIC = 8.16 µM). On the other hand, the in vitro anticancer activity of synthesized analogues **(W1–W21)** were evaluated against the human colorectal carcinoma [HCT116 (ATCC CCL-247)] cancer cell line using the SRB assay and in comparison to 5-FU, the standard drug. Table 1 shows the results in vitro anticancer activity of the synthesized analogues **(W1–W21)**. The anticancer screening results showed that 4-methoxy substituted scaffold i.e. compound **W17** (IC_50_ = 4.12 µM) had the highest anticancer activity amongst the synthesized ones. It was found to be more potent than the standard drug, 5-FU (IC_50_ = 7.69 µM).

**Table 1:** Antimicrobial and anticancer screening results of synthesized analogues

Compounds	Minimum inhibitory concentration (MIC = μM)					IC_50_ (μM)
	Bacterial species			Fungal species		Cancer cell line (HCT116)
	Gram +ve	Gram −ve				
	*SA*	*ST*	*KP*	*CA*	*AN*	
W1	10.15	10.15	10.15	5.08	5.08	> 20.31
W2	11.17	11.17	11.17	5.59	5.59	> 22.35
W3	11.17	11.17	11.17	5.59	5.59	> 22.35
W4	11.17	11.17	11.17	5.59	5.59	> 22.35
W5	10.39	10.39	10.39	5.19	5.19	> 20.77
W6	5.19	5.19	5.19	5.19	5.19	> 20.75
W7	11.44	11.44	11.44	5.72	5.72	> 22.89
W8	11.44	11.44	11.44	11.44	5.72	> 22.89
W9	11.61	11.61	11.61	11.61	5.81	> 23.23
W10	11.61	11.61	11.61	5.81	5.81	> 23.23
W11	12.00	12.00	12.00	6.00	6.00	> 24.01
W12	10.38	10.38	10.38	10.38	5.19	7.08
W13	11.44	11.44	11.44	5.72	5.72	> 22.89
W14	12.42	12.42	12.42	12.42	6.21	> 24.84
W15	11.56	11.56	11.56	11.56	5.78	23.12
W16	11.56	11.56	11.56	11.56	5.78	> 23.12
W17	11.56	11.56	11.56	11.56	5.78	4.12
W18	11.89	11.89	11.89	11.89	5.95	> 23.78
W19	11.89	11.89	11.89	11.89	5.95	> 23.78
W20	11.61	11.61	11.61	11.61	5.81	11.61
W21	10.39	10.39	10.39	10.39	5.19	> 20.77
DMSO	NA	NA	NA	NA	NA	> 1.44
Broth control	NG	NG	NG	NG	NG	–
Ofloxacin	1.73	1.73	1.73	–	–	–
Fluconazole	–	–	–	8.16	8.16	–
5-Florouracil	–	–	–	–	–	7.69

SA: *Staphylococcus aureus* (MTCC3160); ST: *Salmonella typhi* (MTCC3231); KP: *Klebsiella pneumonia* (MTCC9024); CA: *Candida albicans* (MTCC281) and AN: *Aspergillus niger* (MTCC227); DMSO: dimethyl sulfoxide; NA: no activity; NG: no growth

### SAR (structure activity relationship) studies

**Fig. 5:**
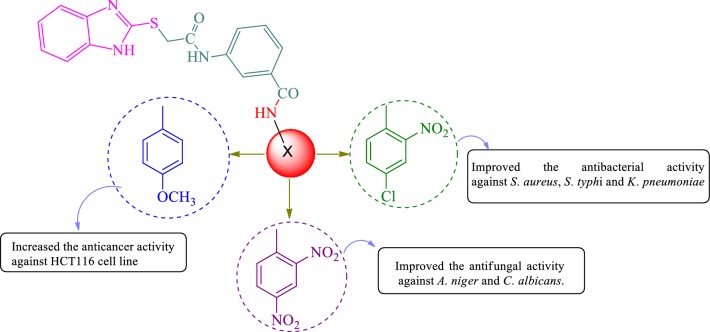
Structural requirements for the antimicrobial and anticancer activities of synthesized benzimidazole analogues

The structure activity relationship for antimicrobial and anticancer screening results of synthesized benzamides (SAR, Fig. 5): substitution of aromatic ring with 2, 4-dinitro substituent (compound **W1**, MIC = 5.08 µM) considerably improved the antifungal activity of benzamide derivatives against *C. albicans* and *A. niger* respectively whereas substitution with 4-chloro-2-nitro group (compound **W6**, MIC = 5.19 µM) resulted in improved antibacterial activity against both Gram-negative *E. coli*, *K. pneumoniae* and Gram-positive *S. aureus* species, respectively. Presence of methoxy group at *para* position of compound **W17** (IC_50_ = 4.12 µM) is responsible for improved antiproliferative activity against HCT116 cancer cell line whereas methoxy group at *ortho* and *meta* position (compound **W15** and **W16,** IC_50_ = 23.12 µM) exhibited lesser activity against same cancer cell line. From the analysis of structures of most active antimicrobial compounds, it may be concluded that the introduction of nitro and halo groups to aromatic ring as an electron-withdrawing moiety may increase the antifungal as well as the antibacterial activities of the synthesized scaffolds. The above findings had demonstrated that variation in nature and position of functional groups on aromatic framework caused a considerable change in anticancer potency. The substitution of methoxy group at *para* position of *N*-phenylbenzamide enhanced the anticancer potential. These studies reveal that different functional groups are required for different activities.

## Experimental section

The reactants and reagents for syntheses were taken from commercial resource. The microbes (Microbial type cell cultures-MTCC) were acquired from Institute of Microbial Technology and Gene bank (IMTECH), Chandigarh. Reaction steps forward was checked by thin layer chromatography (TLC) using ethyl acetate as mobile phase. The scheme was drawn via ChemDraw 8.03. Melting point of synthesized derivatives was determined by open capillary tube technique. An infrared (IR) spectrum was recorded on Bruker 12060280, Software: OPUS 7.2.139.1294 spectrometer using ATR and results were in cm^−1^. Bruker Avance III 600 NMR spectrometer was utilized for ^1^H/^13^C-NMR (DMSO-*d6*, δ ppm). Waters Micromass Q-ToF Micro instrument was used for mass spectra. Elemental analysis was performed on Perkin-Elmer 2400 C, H and N analyzer and all synthesized compounds gave C, H and N analysis within ± 0.4% of the theoretical results.

### *Procedure for synthesized benzamides* (**W1–W21**)

#### *Step a: Synthesis of* int-**I**

A mixture of *m*-aminobenzoic acid (0.01 mol) and triethylamine (0.01 mol) in ethanol was stirred to get a clear solution. Then the solution was cooled in ice for 30 min followed by dropwise addition of chloroacetylchloride (0.01 mol) with stirring (1 h). The resultant precipitate was strained via filtering, desiccated and recrystallized using alcohol [20].

#### *Step b: Synthesis of* int-**II**

To the reaction mixture of 3-(2-chloroacetamido) benzoic acid (int-**I**, 0.01 mol) and 2-mercaptobenzimidazole (0.01 mol) in alcohol potassium carbonate (0.01 mol) was added and then refluxed for 5–6 h and cooled to room temperature followed by evaporation to dryness. The resultant residue was washed with water and recrystallized from ethanol [1].

#### *Step c: Synthesis of* int-**III**

A mixture of thionyl chloride (0.3 mol) and 3-(2-(1*H*-benzo[*d*]imidazol-2-ylthio)acetamido) benzoic acid (int-**II**, 0.25 mol) was refluxed for 2 h. The excess of thionyl chloride was removed by distillation [21].

#### *Step d: Synthesis of final* (**W1–W21**) *benzamides*

The reaction mixture of 3-(2-(1*H*-benzo[*d*]imidazol-2-ylthio)acetamido)benzoyl chloride (int-**III**, 0.01 mol) and substituted aniline (0.01 mol) in suitable solvent was refluxed for appropriate time and thin layer chromatography was used to monitor the reaction. After completion of reaction, it was poured into ice cold water and the resultant precipitate was filtered, desiccated and recrystallized using ethanol [22].

Spectral data elucidation of the synthesized *N*-substituted phenyl benzamide derivatives.

### 3-(2-(1H-benzo[d]imidazol-2-ylthio)acetamido)-N-(2,4-dinitrophenyl)benzamide **(W1)**

M.pt. °C: 200–203; R*f* value: 0.70; % yield: 97.55; IR: [3099 (C–H str.), 1600 (C=C str.) of pn (phenyl nucleus)], 1666 (–CONH str.), 1334 (C=N str.), 1304 (C–N str.), 698 (C–S str., CH_2_-S), 2915 (C–H str., –CH_2_–), 1520 (C–NO_2_ str., C_6_H_5_NO_2_); ^1^H-NMR: 7.16–10.75 (m, 11H, Ar–H), 4.34 (s, 1H, NH of imidazole), 2.53 (s, 2H, CH_2_), 8.15 (s, 2H, (CONH)_2_); ^13^C-NMR: 36.19, 119.67, 119.78, 121.52, 123.06, 123.26, 128.59, 129.00, 129.15, 135.06, 139.015, 149.71, 149.74, 166.38, 167.02; Mol. Formula: C_22_H_16_N_6_O_6_S; Elem. Anal. Calcd: C, 53.66; H, 3.27; N, 17.07; Found: C, 53.69; H, 3.23; N, 17.03; MS: *m/z* 493 [M^+^ +1].

### 3-(2-(1H-benzo[d]imidazol-2-ylthio)acetamido)-N-(2-nitrophenyl)benzamide **(W2)**

M.pt. °C: 212–215; R*f* value: 0.54; % yield: 67.71; IR: [3097 (C–H str.), 1598 (C=C str.) of pn], 1664 (-CONH str., amide), 1335 (C=N str., N=CH), 1304 (C–N str.), 682 (C–S str., CH_2_-S), 2915 (C–H str., –CH_2_–), 1508 (C–NO_2_ str., C_6_H_5_NO_2_); ^1^H-NMR: 7.17–8.30 (m, 12H, Ar–H), 4.34 (s, 1H, NH of imidazole), 2.53 (s, 2H, CH_2_), 7.84 (s, 2H, (CONH)_2_); ^13^C-NMR: 36.18, 115.42, 119.71, 119.81, 121.58, 123.15, 124.28, 125.33, 129.06, 131.37, 135.62, 139.03, 146.14, 166.41, 167.04; Mol. Formula: C_22_H_17_N_5_O_4_S; Elem. Anal. Calcd: C, 59.05; H, 3.83; N, 15.65; Found: C, 53.09; H, 3.87; N, 15.69; MS: *m/z* 448 [M^+^ +1].

### 3-(2-(1H-benzo[d]imidazol-2-ylthio)acetamido)-N-(3-nitrophenyl)benzamide **(W3)**

M.pt.  °C: 213–216; R*f* value: 0.57; % yield: 71.07; IR: [3100 (C–H str.), 1598 (C=C str.) of pn], 1665 (–CONH str., amide), 1335 (C=N str., N=CH), 1304 (C–N str.), 681 (C–S str., CH_2_-S), 2915 (C–H str., –CH_2_–), 1506 (C–NO_2_ str., C_6_H_5_NO_2_); ^1^H-NMR: 7.16–8.32 (m, 12H, Ar–H), 4.36 (s, 1H, NH of imidazole), 2.53 (s, 2H, CH_2_), 7.85 (s, 2H, (CONH)_2_); ^13^C-NMR: 36.20, 113.84, 113.86, 113.87, 121.63, 123.17, 124.29, 129.05, 131.38, 139.03, 149.73, 166.39, 167.05; Mol. Formula: C_22_H_17_N_5_O_4_S; Elem. Anal. Calcd: C, 59.05; H, 3.83; N, 15.65; Found: C, 59.01; H, 3.79; N, 15.61 MS: *m/z* 448 [M^+^ +1].

### 3-(2-(1H-benzo[d]imidazol-2-ylthio)acetamido)-N-(4-nitrophenyl)benzamide **(W4)**

M.pt.  °C: 206–209; R*f* value: 0.69; % yield: 78.25; IR: [3100 (C–H str.), 1595 (C=C str.) of pn], 1662 (–CONH str., amide), 1331 (C=N str., N=CH), 1302 (C–N str.), 679 (C–S str., CH_2_-S), 2913 (C–H str., –CH_2_–), 1554 (C–NO_2_ str., C_6_H_5_NO_2_); ^1^H-NMR: 7.19–8.33 (m, 12H, Ar–H), 4.37 (s, 1H, NH of imidazole), 2.54 (s, 2H, CH_2_), 8.01 (s, 2H, (CONH)_2_); ^13^C-NMR: 36.21, 113.94, 119.84, 121.67, 123.17, 124.31, 126.33, 129.05, 139.03, 139.12, 139.14, 149.73, 166.39, 167.06; Mol. Formula: C_22_H_17_N_5_O_4_S; Elem. Anal. Calcd: C, 59.05; H, 3.83; N, 15.65; Found: C, 59.08; H, 3.87; N, 15.61; MS: *m/z* 448 [M^+^ +1].

### 3-(2-(1H-benzo[d]imidazol-2-ylthio)acetamido)-*N*-(4-bromophenyl)benzamide **(W5)**

M.pt.  °C: 202–205; R*f* value: 0.72; % yield: 73.96; IR: [3107 (C–H str.), 1597 (C=C str.) of pn], 1665 (–CONH str., amide), 1334 (C=N str., N=CH), 1304 (C–N str.), 681 (C–S str., CH_2_-S), 2915 (C–H str., –CH_2_–), 651 (C–Br str., C_6_H_5_Br); ^1^H-NMR: 7.18–8.33 (m, 12H, Ar–H), 4.36 (s, 1H, NH of imidazole), 4.34 (s, 2H, CH_2_), 7.86 (s, 2H, (CONH)_2_); ^13^C-NMR: 36.20, 119.84, 121.60, 123.17, 124.30, 129.05, 131.38, 139.03, 149.73, 166.41, 1667.06; Mol. Formula: C_22_H_17_N_4_O_2_SBr; Elem. Anal. Calcd: C, 54.89; H, 3.56; N, 11.64; Found: C, 54.84; H, 3.52; N, 11.60; MS: *m/z* 482 [M^+^ +1].

### 3-(2-(1H-benzo[d]imidazol-2-ylthio)acetamido)-N-(2-nitro-4-chlorophenyl)benzamide **(W6)**

M.pt.  °C: 215–218; R*f* value: 0.76; % yield: 97.71; IR: [3100 (C–H str.), 1599 (C=C str.) of pn], 1670 (–CONH str., amide), 1367 (C=N str., N=CH), 1338 (C–N str.), 681 (C–S str., CH_2_-S), 2915 (C–H str., –CH_2_–),758 (C–Cl str., C_6_H_5_Cl), 1503 (C–NO_2_ str., C_6_H_5_NO_2_); ^1^H-NMR: 7.16–8.29 (m, 11H, Ar–H), 4.33 (s, 1H, NH of imidazole), 7.96 (s, 2H, (CONH)_2_); ^13^C-NMR: 36.18, 118.30, 121.15, 123.13, 124.00, 124.26, 129.97, 131.36, 139.03, 145.03, 166.40, 167.02; Mol. Formula: C_22_H_16_N_5_O_4_SCl; Elem. Anal. Calcd: C, 54.83; H, 3.35; N, 14.53; Found: C, 54.87; H, 3.39; N, 14.57; MS: *m/z* 482 [M^+^ +1].

### 3-(2-(1H-benzo[d]imidazol-2-ylthio)acetamido)-N-(2-chlorophenyl)benzamide **(W7)**

M.pt.  °C: 217–220; R*f* value: 0.44; % yield: 89.98; IR: [3098 (C–H str.), 1598 (C=C str.) of pn], 1664(-CONH str., amide), 1335 (C=N str., N=CH), 1304 (C–N str.), 681 (C–S str., CH_2_-S), 2915 (C–H str., –CH_2_-), 742 (C–Cl str., C_6_H_5_Cl); ^1^H-NMR: 7.14–8.33 (m, 12H, Ar–H), 4.33 (s, 1H, NH of imidazole), 7.84 (s, 2H, (CONH)_2_); ^13^C-NMR: 36.18, 119.80, 119.78, 121.50, 123.15, 124.28, 129.06, 131.37, 139.04, 149.71, 166.42, 167.04; Mol. Formula: C_22_H_17_N_4_O_2_SCl; Elem. Anal. Calcd: C, 60.48; H, 3.92; N, 12.82; Found: C, 60.44; H, 3.96; N, 12.86; MS: *m/z* 437 [M^+^ +1].

### 3-(2-(1H-benzo[d]imidazol-2-ylthio)acetamido)-N-(3-chlorophenyl)benzamide **(W8)**

M.pt.  °C: 219–222; R*f* value: 0.66; % yield: 77.47; IR: [3097 (C–H str.), 1608 (C=C str.) of pn], 1665 (–CONH str., amide), 1369 (C=N str., N=CH), 1338 (C–N str.), 688 (C–S str., CH_2_-S), 2945 (C–H str., –CH_2_–), 745 (C–Cl str., C_6_H_5_Cl); ^1^H-NMR: 7.15–8.31 (m, 12H, Ar–H), 4.35 (s, 1H, NH of imidazole), 7.85 (s, 2H, (CONH)_2_); ^13^C-NMR: 36.18, 119.81, 119.78, 121.51, 123.16, 124.29, 129.06, 131.38, 139.04, 149.71, 166.43, 167.05; Mol. Formula: C_22_H_17_N_4_O_2_SCl; Elem. Anal. Calcd: C, 60.48; H, 3.92; N, 12.82; Found: C, 60.52; H, 3.88; N, 12.85; MS: *m/z* 437 [M^+^ +1].

### 3-(2-(1H-benzo[d]imidazol-2-ylthio)acetamido)-N-(2,6-dimethylphenyl)benzamide **(W9)**

M.pt.  °C: 218–221; R*f* value: 0.51; % yield: 77.62; IR: [3100 (C–H str.), 1599 (C=C str.) of pn], 1665 (–CONH str., amide), 1336 (C=N str., N=CH), 1304 (C–N str.), 704 (C–S str., CH_2_-S), 2915 (C–H str., –CH_2_–), 2886 (C–H str., CH_3_); ^1^H-NMR: 7.14–8.30 (m, 11H, Ar–H), 4.33 (s, 1H, NH of imidazole), 3.87 (s, 2H, CH_2_), 7.84 (s, 2H, (CONH)_2_), 2.52 (s, 6H, (CH_3_)_2_); ^13^C-NMR: 36.17, 119.80, 121.50, 123.15, 124.27, 129.07, 131.37, 139.04, 149.71, 166.42, 167.04; Mol. Formula: C_24_H_22_N_4_O_2_S; Elem. Anal. Calcd: C, 66.96; H, 5.15; N, 13.01; Found: C, 66.92; H, 5.15; N, 13.01; MS: *m/z* 431 [M^+^ +1].

### 3-(2-(1H-benzo[d]imidazol-2-ylthio)acetamido)-N-(2,4-dimethylphenyl)benzamide **(W10)**

M.pt.  °C: 220–223; R*f* value: 0.58; % yield: 55.02; IR: [3098 (C–H str.), 1598 (C=C str.) of pn], 1664 (-CONH str., amide), 1336 (C=N str., N=CH), 1302 (C–N str.), 704 (C–S str., CH_2_-S), 2912 (C–H str., –CH_2_–), 2884 (C–H str., CH_3_); ^1^H-NMR: 7.13–8.27 (m, 11H, Ar–H), 4.31 (s, 1H, NH of imidazole), 2.52 (s, 2H, CH_2_), 7.82 (s, 2H, (CONH)_2_), 2.51 (s, 6H, (CH_3_)_2_); ^13^C-NMR: 36.16, 119.80, 119.78, 121.49, 123.14, 124.26, 124.32, 129.07, 131.36, 139.04, 149.70, 166.40, 167.02; Mol. Formula: C_24_H_22_N_4_O_2_S; Elem. Anal. Calcd: C, 66.96; H, 5.15; N, 13.01; Found: C, 66.99; H, 5.11; N, 13.04; MS: *m/z* 431 [M^+^ +1].

### 3-(2-(1H-benzo[d]imidazol-2-ylthio)acetamido)-N-(3-methylphenyl)benzamide **(W11)**

M.pt.  °C: 210–213; R*f* value: 0.50; % yield: 91.05; IR: [3105 (C–H str.), 1608 (C=C str.) of pn], 1663 (-CONH str., amide), 1372 (C=N str., N=CH), 1334 (C–N str.), 708 (C–S str., CH_2_-S), 2913 (C–H str., –CH_2_–), 2869 (C–H str., CH_3_); ^1^H-NMR: 7.16–8.33 (m, 12H, Ar–H), 4.37 (s, 1H, NH of imidazole), 7.86 (s, 2H, (CONH)_2_); ^13^C-NMR: 36.19, 113.80, 113.82, 119.83, 121.59, 123.17, 124.30, 129.05, 131.38, 139.04, 149.73, 166.42, 167.06; Mol. Formula: C_23_H_20_N_4_O_2_S; Elem. Anal. Calcd: C, 66.33; H, 4.84; N, 13.45; Found: C, 66.36; H, 4.88; N, 13.49; MS: *m/z* 417 [M^+^ +1].

### 3-(2-(1H-benzo[d]imidazol-2-ylthio)acetamido)-N-(2-chloro-4-nitrophenyl)benzamide **(W12)**

M.pt.  °C: 207–210; R*f* value: 0.56; % yield: 93.92; IR: [3098 (C–H str.), 1598 (C=C str.) of pn], 1665 (-CONH str., amide), 1321 (C=N str., N=CH), 1305 (C–N str.), 681 (C–S str., CH_2_-S), 2842 (C–H str., –CH_2_–), 743 (C–Cl str., C_6_H_5_Cl), 1484 (C–NO_2_ str., C_6_H_5_NO_2_); ^1^H-NMR: 7.19–8.31 (m, 11H, Ar–H), 4.37 (s, 1H, NH of imidazole), 7.99 (s, 2H, (CONH)_2_); ^13^C-NMR: 36.22, 113.81, 115.51, 119.83, 123.16, 124.29, 124.56, 125.58, 129.03, 131.37, 135.90, 139.01, 149.75, 151.29, 166.32, 167.04; Mol. Formula: C_22_H_16_N_5_O_4_SCl; Elem. Anal. Calcd C, 54.83; H, 3.35; N, 14.53; Found: C, 54.87; H, 3.38; N, 14.57; MS: *m/z* 482 [M^+^ +1].

### 3-(2-(1H-benzo[d]imidazol-2-ylthio)acetamido)-N-(4-chlorophenyl)benzamide **(W13)**

M.pt.  °C: 206–209; R*f* value: 0.45; % yield: 79.12; IR: [3105 (C–H str.), 1598 (C=C str.) of pn], 1665 (-CONH str., amide), 1334 (C=N str., N=CH), 1304 (C–N str.), 681 (C–S str., CH_2_-S), 2911 (C–H str., –CH_2_–), 742 (C–Cl str., C_6_H_5_Cl); ^1^H-NMR: 7.18–8.34 (m, 12H, Ar–H), 4.37 (s, 1H, NH of imidazole), 4.35 (s, 2H, CH_2_), 7.87 (s, 2H, (CONH)_2_); ^13^C-NMR: 36.21, 113.90, 119.84, 123.12, 123.17, 124.31, 128.68, 129.05, 137.75, 139.03, 139.10, 149.73, 166.27, 167.06; Mol. Formula: C_22_H_17_N_4_O_2_SCl; Elem. Anal. Calcd. C, 60.48; H, 3.92; N, 12.82; Found: C, 60.44; H, 3.96; N, 12.86; MS: *m/z* 437 [M^+^ +1].

### 3-(2-(1H-benzo[d]imidazol-2-ylthio)acetamido)-N-(phenyl)benzamide **(W14)**

M.pt.  °C: 203–206; R*f* value: 0.45; % yield: 99.18; IR: [3093 (C–H str.), 1598 (C=C str.) of pn], 1663 (-CONH str., amide), 1334 (C=N str., N=CH), 1304 (C–N str.), 703 (C–S str., CH_2_-S), 2910 (C–H str., –CH_2_–); ^1^H-NMR: 7.13–8.29 (m, 13H, Ar–H), 4.32 (s, 1H, NH of imidazole), 7.83 (s, 2H, (CONH)_2_); ^13^C-NMR: 36.19, 119.82, 121.52, 123.16, 124.29, 129.06, 131.38, 139.04, 149.72, 166.44, 167.06; Mol. Formula: C_22_H_18_N_4_O_2_S; Elem. Anal. Calcd. C, 65.65; H, 4.51; N, 13.92; Found: C, 65.69; H, 4.55; N, 13.96; MS: *m/z* 403 [M^+^ +1].

### 3-(2-(1H-benzo[d]imidazol-2-ylthio)acetamido)-N-(4-methoxyphenyl)benzamide **(W15)**

M.pt.  °C: 204–207; R*f* value: 0.70; % yield: 84.45; IR: [3088 (C–H str.), 1598 (C=C str.) of pn], 1664 (-CONH str., amide), 1335 (C=N str., N=CH), 1303 (C–N str.), 681 (C–S str., CH_2_-S), 2914 (C–H str., –CH_2_–), 1200 (C–O–C str., phenyl ether), 2832 (C–H str., O-CH_3_); ^1^H-NMR: 7 7.15–8.32 (m, 12H, Ar–H), 4.35 (s, 1H, NH of imidazole), 7.85 (s, 2H, (CONH)_2_), 2.53 (s, 3H, OCH_3_); ^13^C-NMR: 36.18, 119.82, 121.52, 123.16, 124.29, 129.06, 131.38, 139.04, 149.72, 166.44, 167.06; Mol. Formula: C_23_H_20_N_4_O_3_S; Elem. Anal. Calcd. C, 63.87; H, 4.66; N, 12.95; Found: C, 63.85; H, 4.69; N, 12.98; MS: *m/z* 433 [M^+^ +1].

### 3-(2-(1H-benzo[d]imidazol-2-ylthio)acetamido)-N-(3-methoxyphenyl)benzamide **(W16)**

M.pt.  °C: 209–212; R*f* value: 0.72; % yield: 81.90; IR: [3100 (C–H str.), 1598 (C=C str.) of pn], 1665 (-CONH str., amide), 1336 (C=N str., N=CH), 1304 (C–N str.), 704 (C–S str., CH_2_-S), 2913 (C–H str., –CH_2_–), 1201 (C–O–C str., phenyl ether), 2820 (C–H str., O-CH_3_); ^1^H-NMR: 7.16–8.33 (m, 12H, Ar–H), 4.36 (s, 1H, NH of imidazole), 7.86 (s, 2H, (CONH)_2_); ^13^C-NMR: 36.18, 113.75, 119.82, 121.15, 123.16, 124.29, 129.06, 131.38, 139.04, 149.72, 166.44, 167.06; Mol. Formula: C_23_H_20_N_4_O_3_S; Elem. Anal. Calcd. C, 63.87; H, 4.66; N, 12.95; Found: C, 63.85; H, 4.62; N, 12.99; MS: *m/z* 433 [M^+^ +1].

### 3-2-(1H-benzo[d]imidazol-2-ylthio)acetamido)-N-(4-methoxyphenyl)benzamide** (W17)**

M.pt.  °C: 214–217; R*f* value: 0.45; % yield: 79.81; IR: [3100 (C–H str.), 1598 (C=C str.) of pn], 1664 (-CONH str., amide), 1336 (C=N str., N=CH), 1304 (C–N str.), 703 (C–S str., CH_2_-S), 2914 (C–H str., –CH_2_–), 1201 (C–O–C str., phenyl ether), 2830 (C–H str., O-CH_3_); ^1^H-NMR: 7.14–8.30 (m, 12H, Ar–H), 4.34 (s, 1H, NH of imidazole), 2.52 (s, 2H, CH_2_), 7.84 (s, 2H, (CONH)_2_), 3.73 (s, 3H, OCH_3_); ^13^C-NMR: 36.08, 55.10, 113.90, 114.60, 119.80, 121.49, 123.15, 124.28, 129.06, 131.37, 131.97, 139.04, 139.23, 149.71, 155.34, 165.61, 167.04; Mol. Formula C_23_H_20_N_4_O_3_S; Elem. Anal. Calcd. C, 63.87; H, 4.66; N, 12.95; Found: C, 63.91; H, 4.69; N, 12.91; MS: *m/z* 433 [M^+^ +1].

### 3-(2-(1H-benzo[d]imidazol-2-ylthio)acetamido)-N-(2-florophenyl)benzamide **(W18)**

M.pt.  °C: 216–219; R*f* value: 0.42; % yield: 80.86; IR: [3105 (C–H str.), 1599 (C=C str.) of pn], 1665 (–CONH str., amide), 1335 (C=N str., N=CH), 1304 (C–N str.), 681 (C–S str., CH_2_-S), 2916 (C–H str., –CH_2_–), 1085 (C-F str., C_6_H_5_F); ^1^H-NMR: 7.12–8.26 (m, 12H, Ar–H), 4.31 (s, 1H, NH of imidazole), 2.51 (s, 2H, CH_2_), 7.84 (s, 2H, (CONH)_2_); ^13^C-NMR: 36.16, 119.77, 121.51, 123.13, 124.25, 129.07, 131.36, 139.03, 149.70, 166.39, 167.01; Mol. Formula C_22_H_17_N_4_O_2_SF; Elem. Anal. Calcd. C, 62.84; H, 4.08; N, 13.33; Found: C, 62.88; H, 4.04; N, 13.29; MS: *m/z* 421 [M^+^ +1].

### 3-(2-(1H-benzo[d]imidazol-2-ylthio)acetamido)-N-(4-florophenyl)benzamide **(W19)**

M.pt.  °C: 207–210; R*f* value: 0.51; % yield: 70.64; IR: [3094 (C–H str.), 1598 (C=C str.) of pn], 1665 (–CONH str., amide), 1334 (C=N str., N=CH), 1304 (C–N str.), 704 (C–S str., CH_2_-S), 2915 (C–H str., –CH_2_–), 1084 (C-F str., C_6_H_5_F); ^1^H-NMR: 7.16–8.32 (m, 12H, Ar–H), 4.36 (s, 1H, NH of imidazole), 7.86 (s, 2H, (CONH)_2_); ^13^C-NMR: 36.18, 119.82, 121.60, 123.16, 124.29, 129.05, 131.37, 139.02, 149.72, 166.40, 167.04; Mol. Formula C_22_H_17_N_4_O_2_SF; Elem. Anal. Calcd. C, 62.84; H, 4.08; N, 13.33; Found: C, 62.81; H, 4.12; N, 13.37; MS: *m/z* 421 [M^+^ +1].

### 3-(2-(1H-benzo[d]imidazol-2-ylthio)acetamido)-N-ethyl-N-phenylbenzamide **(W20)**

M.pt.  °C: 216–219; R*f* value: 0.42; % yield: 83.22; IR: [3096 (C–H str.), 1598 (C=C str.) of pn], 1664 (–CONH str., amide), 1336 (C=N str., N=CH), 1304 (C–N str.), 701 (C–S str., CH_2_-S), 2915 (C–H str., –CH_2_–), 2932 (C–H str., CH_3_), 2826 (C–H str., N-CH_3_); ^1^H-NMR: 7.12–8.33 (m, 13H, Ar–H), 4.31 (s, 1H, NH of imidazole), 7.82 (s, 2H, (CONH)_2_), 2.51 (q, 2H, CH_2_); ^13^C-NMR: 36.16, 119.78, 119.78, 121.49, 123.14, 124.26, 129.07, 131.37, 139.04, 149.70, 166.40, 167.02; Mol. Formula C_24_H_22_N_4_O_2_S; Elem. Anal. Calcd. C, 66.96; H, 5.15; N, 13.01; Found C, 66.93; H, 5.19; N, 13.04; MS: *m/z* 431 [M^+^ +1].

### 3-(2-(1H-benzo[d]imidazol-2-ylthio)acetamido)-N-(3-bromophenyl)benzamide **(W21)**

M.pt.  °C: 221–223; R*f* value: 0.47; % yield: 70; IR: [3099 (C–H str.), 1598 (C=C str.) of pn], 1665 (–CONH str., amide), 1336 (C=N str., N=CH), 1304 (C–N str.), 704 (C–S str., CH_2_-S), 2915 (C–H str., –CH_2_–), 653 (C–Br str., Br); ^1^H-NMR: 7.15–8.29 (m, 12H, Ar–H), 4.33 (s, 1H, NH of imidazole), 2.52 (s, 2H, CH_2_), 7.83 (s, 2H, (CONH)_2_); ^13^C-NMR: 36.17, 119.80, 119.80, 121.50, 123.15, 124.27, 129.06, 131.37, 139.04, 149.70, 166.41, 167.03; Mol. Formula C_22_H_17_N_4_O_2_SBr; Elem. Anal. Calcd. C, 54.89; H, 3.56; N, 11.64; Found C, 54.85; H, 3.59; N, 11.68; MS: *m/z* 482 [M^+^ +1].

### Biological studies

#### Antimicrobial evaluation

The in vitro antimicrobial potential of the synthesized benzamides **(W1–W21)** in μM was determined against Gram-positive bacteria (*Staphylococcus aureus*- MTCC 3160), Gram-negative bacterium (*Klebsiella pneumoniae*- MTCC 9024, *Salmonella typhi*- MTCC 3231) and fungal strains (*Aspergillus niger*- MTCC 281 and *Candida albicans*-MTCC 227) by tube dilution method [23] using ofloxacin (antibacterial) and fluconazole (antifungal) as standard. DMSO was used to dissolve the reference and experimental molecules **(W1–W21)**. Dilutions were set up in nutrient broth (I.P.) for bacterial (incubated at 37 ± 1 °C for 24 h) and Sabouraud dextrose broth (I.P.) for fungal species (25 ± 1 °C for 7 days for *A. niger*) and (37 ± 1 °C for 48 h for *C. albicans*) [24].

#### Anticancer evaluation

The in vitro anticancer potential of the synthesized substituted benzamides was determined against the human colorectal cancer cell line using the SRB assay [25]. HCT116 cells were seeded onto the wells of 96-mL plates at 2500 cells/well for 24 h. The reference and test drug molecules were dissolved in DMSO subjected to serial dilutions. They were then incubated with monolayer cells at 37 °C for 72 h. the treated cells were then fixed with trichloroacetic acid for and then stained with 0.4% *w/v* of SRB in acetic acid. The unbound stain was removed by washing with 1% acetic acid. Bound SRB was solubilised in 10 mM Tris base solution. Absorbance was measured by a computer-interfaced 96-well plate spectrophotometer at 570 nm.

## Conclusion

The synthetic work was conducted under appropriate experimental conditions and the expected compounds had been obtained. The biological studies were carried out to observe the effect of substituents on the antimicrobial and anticancer activities. From the outcomes of antimicrobial and anticancer studies, it is concluded that the introduction of electron-withdrawing groups such as nitro and halo substituents increased the antimicrobial potential of the synthesized scaffolds *i.e.* compounds **W1** (3-(2-(1*H*-benzo[*d*]imidazol-2-ylthio)acetamido)-*N*-(2,4-dinitrophenyl)benzamide) and **W6** (3-(2-(*1*H-benzo[*d*]imidazol-2-ylthio)acetamido)-*N*-(2-nitro-4-chlorophenyl)benzamide) while the introduction of *para*-methoxy substituent to phenyl ring as an electron-donating group *i.e.* compound **W17** (3-(2-(1*H*-benzo[*d*]imidazol-2-ylthio)acetamido)-*N*-(4-methoxyphenyl)benzamide) exhibited the most promising anticancer activity amongst all tested compounds.
